# Supplementary material for: Incidence and predictors of mortality within the first year of antiretroviral therapy initiation at Debre-Markos Referral Hospital, Northwest Ethiopia: A retrospective follow up study
Source: PLoS One. 2021 May 14;16(5):e0251648. doi: 10.1371/journal.pone.0251648 (PMC8121335; doi:10.1371/journal.pone.0251648)
Supplement: S1 Table — (DOCX) [file pone.0251648.s001.docx]

**S1 Table:** Bi-variable Cox regression analysis of predictors of mortality within one year of ART initiation among adults on ART at Debre-Markos Referral Hospital; Jan 1, 2014 to Dec 31, 2018 (n = 494)

| **Characteristics** | **Outcome** | | **CHR (95% CI)** | **P-value** |
| --- | --- | --- | --- | --- |
|  | **Died** | **Censored** |  |  |
| Patient’s sex |  |  |  |  |
| Male | 24 | 167 | 1.00 |  |
| Female | 30 | 273 | 0.74 (0.43, 1.26) | 0.269 |
| Patient’s Age in years |  |  |  |  |
| 15 - 24 | 4 | 57 | 1.00 |  |
| 25 - 34 | 21 | 179 | 1.68 (0.58, 4.90) | 0.340 |
| 35 - 44 | 19 | 141 | 1.93 (0.66, 5.68) | 0.231 |
| ≥ 45 | 10 | 63 | 2.16 (0.68, 6.89) | 0.192* |
| Level of education |  |  |  |  |
| No education | 17 | 154 | 0.71 (0.39, 1.29) | 0.256 |
| 1^0^ education | 8 | 105 | 0.50 (0.23, 1.09) | 0.083* |
| 2^0^ & above | 29 | 181 | 1.00 |  |
| Patient’s Occupation |  |  |  |  |
| Employed | 22 | 141 | 1.00 |  |
| Un employed | 32 | 299 | 0.67 (0.39, 1.15) | 0.144* |
| Patient’s Residence |  |  |  |  |
| Rural | 16 | 90 | 1.65 (0.92, 2.95) | 0.094* |
| Urban | 38 | 350 | 1.00 |  |
| Patient Household number |  |  |  |  |
| ≤ 2 person | 26 | 171 | 1.00 |  |
| >2 persons | 28 | 269 | 0.69 (0.41, 1.18) | 0.178* |
| HIV disclosure status |  |  |  |  |
| Disclosed | 47 | 403 | 1.00 |  |
| Not disclosed | 7 | 37 | 1.50 (0.68, 3.33) | 0.314 |
| Pre-ART duration |  |  |  |  |
| < 6 months | 29 | 321 | 1.00 |  |
| ≥ 6 months | 25 | 119 | 2.07 (1.21, 3.53) | 0.008* |
| Baseline Anemia |  |  |  |  |
| Yes | 23 | 143 | 1.66 (0.97, 2.85) | 0.066* |
| No | 31 | 297 | 1.00 |  |
| Past opportunistic infection |  |  |  |  |
| Yes | 7 | 72 | 0.77 (0.35, 1.69) | 0.513 |
| No | 47 | 368 |  |  |
| Body mass index |  |  |  |  |
| <18.5 kg/m^2^ | 17 | 120 | 1.29 (0.72, 2.33) | 0.182* |
| 18.5-24.9 kg/m^2^ | 33 | 269 | 1.00 |  |
| > 24.9 kg/m^2^ | 4 | 51 | 0.66 (0.24,1.87) | 0.440 |
| Baseline clinical Staging |  |  |  |  |
| I/II | 33 | 306 | 1.00 |  |
| III | 16 | 110 | 1.36 (0.75, 2.48) | 0.308 |
| IV | 5 | 24 | 2.09 (0.82, 5.36) | 0.124* |
| Baseline CD4 count (cells/μl) |  |  |  |  |
| < 100 | 10 | 79 | 1.06 (0.51, 2.24) | 0.870 |
| 100-199 | 7 | 67 | 0.82 (0.35, 1.91) | 0.649 |
| 200-349 | 14 | 110 | 0.99 (0.51, 1.92) | 0.967 |
| ≥ 350 | 23 | 184 | 1.00 |  |
| Baseline functional status |  |  |  |  |
| Working | 36 | 356 | 1.00 |  |
| Ambulatory/bedridden | 18 | 84 | 2.31 (1.31, 4.07) | 0.004* |
| Took IPT during follow up |  |  |  |  |
| Yes | 13 | 182 | 1.00 |  |
| No | 41 | 258 | 2.28 (1.22, 4.25) | 0.01* |
| Took CPT at follow up |  |  |  |  |
| Yes | 32 | 289 | 1.00 |  |
| No | 22 | 151 | 1.48 (0.86, 2.54) | 0.160* |
| TB Co-infection |  |  |  |  |
| Yes | 7 | 36 | 2.35 (1.06, 5.22) | 0.036* |
| No | 47 | 404 | 1.00 |  |

**Variables significant with bi-variable Cox regression analysis at p-value < 0.2 and entered to multi-variable analysis.*
