# Supplementary material for: Incidence and predictors of mortality within the first year of antiretroviral therapy initiation at Debre-Markos Referral Hospital, Northwest Ethiopia: A retrospective follow up study
Source: PLoS One. 2021 May 14;16(5):e0251648. doi: 10.1371/journal.pone.0251648 (PMC8121335; doi:10.1371/journal.pone.0251648)
Supplement: Attachment — Submitted filename: Point by point response.docx [file pone.0251648.s003.docx]

27/01/2021

PLOS ONE editors

Journal of PLOS ONE

Dear PLOS ONE editors

**Subject**: Submission of revised manuscript entitled as ***“Incidence and predictors of mortality within the first year of antiretroviral therapy initiation at Debre-Markos referral hospital, Northwest Ethiopia: A retrospective follow up study*”**.

EMID: f5e77dd8ff285cde

Thank you for email dated on December 17, 2020, enclosing the reviewer’s comments. We have carefully revised the manuscript and incorporated their comments accordingly. Our responses are given in point-by-point response below.

We hope the revised version is suitable for publication and look forward to hearing from you in due courses.

**Sincerely!**

Agazhe Aemro Terefe (Corresponding author)

University of Gondar, College of Medicine and health Sciences, School of Nursing, Department of Medical Nursing.

**Point by point response to reviewers’ comments**

**Part I: Editors’ concerns and response by the authors:**

1. **Editors’ concern:**

Please ensure that your manuscript meets PLOS ONE's style requirements, including those for file naming

**Authors’ response:**

The authors tried to ensure that the manuscript meets PLOS ONE's style requirements.

2. **Editors’ concern:**

Please provide additional details regarding participant consent.

**Authors’ response:**

This study was based on chart review so that individual consent from each participant is not important, even it is impossible to get individual consent from each participant. There for, the one who is responsible to give the consent for the authors to collect data from the chart was medical director of the hospital. So, the medical director gave to the authors a signed consent and we took it and gave to data clerks so that the data collectors could extract data from the charts. Additionally, all data extracted from patient’s chart were fully anonymized.

3. **Editors’ concern:**

We suggest you thoroughly copyedit your manuscript for language usage, spelling, and grammar.

**Authors’ response:**

With repeated reading and with the help of online grammar checker, the authors tried to correct problems regarding to language usage, spelling, and grammar.

4. **Editors’ concern:**

Please ensure you have thoroughly discussed any potential limitations of this study within the Discussion section, including the potential impact of confounding factors.

**Authors’ response:**

Potential limitations of the study were stated in the revised manuscript next to the discussion part. Additionally, confounding factors were reduced by excluding from multi-variable analysis of those variables with p-value of greater than 0.2 at bi-variable analysis.

5. **Editors’ concern:**

We note that you have indicated that data from this study are available upon request. PLOS only allows data to be available upon request if there are legal or ethical restrictions on sharing data publicly?

**Authors’ response:**

Thank you for your clarification.

The message that I expressed as “data from this study are available upon request” doesn’t mean there is a restriction of availing the data set. Rather it was to mean that the data will be avail based on your interest or your need.

a. **Editors’ concern:**

If there are ethical or legal restrictions on sharing a de-identified data set, please explain them in detail

**Authors’ response:**

In this data set, there are no ethical or legal restrictions on sharing a de-identified data set; because, there is no potentially identified or sensitive patient information and it is fully anonymized.

b. As per the request, minimal anonymized data set is uploaded.
6. **Editors’ concern:**

Your ethics statement should only appear in the Methods section of your manuscript. If your ethics statement is written in any section besides the Methods, please move it to the Methods section and delete it from any other section. Please ensure that your ethics statement is included in your manuscript, as the ethics statement entered into the online submission form will not be published alongside your manuscript

**Authors’ response:**

Thanks for your information; the ethics statement is incorporated only in the Methods section of the revised manuscript.

7. **Editors’ concern:**

We note you have included a table to which you do not refer in the text of your manuscript. Please ensure that you refer to Table 4. in your text; if accepted, production will need this reference to link the reader to the Table.

**Authors’ response:**

Yes, the author accept it and made a correction on the manuscript. Actually there was no any table which the author didn’t refer in the text of the previous manuscript. There were three tables (table 1, table 2, and table 3), of which table 2 was divided in to two pages with a title “table 2 (continued)” and this might resulted in confusion to both the editors and reviewers. By considering this, the authors tried to merge table 2 and put it in a single page of the revised manuscript.

**Part II: Reviewers' comments and response by the authors:**
**Reviewer #1**: The aims of the study are very interesting and appealing. Indeed, the authors explored the impact of a large number of variables on the clinical outcomes. In my opinion, the paper could be more clear and easy to read if results are summarized better, avoiding to duplicate results in table and text. They need to report a valid legend for the KM figures and log Rank p results. Table 4 is outlined.

**Authors’ response:**

- The authors highlighted main results in the text and tried to summarize in some manner, as per the comment. But, it could be clear that not all findings on the table are incorporated in the text.
- Based on the comment the authors give valid legend for the KM figures.
- Both chi-square and log-rank p-values are incorporated in the revised manuscript.
- The authors re-organize the tables as table 1, 2, and 3 by removing “table 2 continued”. Also, the tables are cited immediately after the text they tend to be referred.

**Reviewer #2:**

**General comment by reviewer#2:**

Abbreviations should be written entirely in the first appearance both in abstract and in full text.
Many typo and grammatical mistakes are present in the manuscript. I suggest to include an English mother tongue revisor to check the manuscript.

**Authors’ response:**

Thank you for your comment and I tried to consider all the comments; Abbreviations had written entirely in the first appearance and attempt was made to correct grammatical mistakes present in the manuscript with repeated reading and using of online grammar checker (<https://www.scribens.com/>, <https://www.grammarcheck.net/editor/>).

**Introduction:**

**Reviewer concern:**

The authors wrote "But HIV-infected individuals still hesitates to start the treatment early which results in progression of the disease to the advanced stage. This intern results in risk of early mortality for patients with HIV". Probably the hesitation is due to cultural behavior. I suggest specifying that this sentence concern the African or Ethiopian situation.
About the introduction, I suggest adding a part about the efficacy and tolerability of the new drugs also in the advanced HIV-infected naïve patients.
**Authors’ response:**

- The authors tried to edit the idea regarding the term “hesitation”.
- As per the comment, authors incorporated the effect of Drug discontinuation on the prognosis, on the introduction part.

**Method:**

**Reviewer concern:**

The methods section is quite long. I suggest deleting the geographical part; although it is very interesting, it does not add crucial information.
In the results section, the authors wrote about anemia. I suggest adding the definition of anemia in methods.

**Authors’ Response:**

- Based on the comment, the authors tried to avoid the geographical part in order to shorten the method.
- As per the comment, the authors included the definition of anemia and ART adherence to the method section of the revised manuscript.

**Result and discussion:**

**Reviewer concern**: the authors wrote, "But the 179 death rate was high in males which is 16.32 deaths per 100 person year." I suggest also adding the number of female deaths per 100 person-years to make clear the difference

**Authors’ Response:**

- We would like to say thank you for the comments. You ask the author to add number of ‘***female deaths per 100 person-years’*** to make clear the difference. Even though it was stated in Table one of the manuscript, the authors incorporated it to the text part of the revised manuscript.

**Reviewer concern**: Also they should define what they intend with good, fair, and poor adherence to antiretroviral treatment.

**Authors’ Response:**

- As stated above, the definition of ART drug adherence was incorporated to the method section of the revised manuscript.

**Reviewer concern**: No data about baseline HIV-RNA load is present. I believe that this information is crucial both for deaths and virological failure. If this information is not available, the authors should discuss it in the limit's section.

**Authors’ Response:**

- The reviewer also asked the author to include data about baseline HIV-RNA load and it is true that baseline HIV-RNA load is crucial both for deaths and virologic failure.
- But, information related to viral load was not found on the majority of patients’ charts.
- By this reason we were unable to incorporate it to our study; so, based on your suggestion we stated it as a limitation in the revised manuscript.

**Reviewer concern**: The authors wrote, "From these, pre-ART duration, residence, functional status at baseline, and took Cotrimoxazole preventive therapy during follow up were found to be statistically significant predictors of the death rate among adults within the first year of ART initiation at a P-value of less than 0.05 (Table 3)". I think the authors mean "table 4". Check it?

**Authors’ Response:**

- Actually it is table 3 not table 4 and the problem is on table 2 that I divided it to two by using the term “**Table 2 (continued)**”; but, in the revised manuscript table 2 is merged to a single table and the regression table remains as it is (i.e. table 3).

**Reviewer concern**:

- No data about the cause of death are present. I suggest adding them.
- The presence of opportunistic infections at the moment of diagnosis is not present in the Cox model. Which was the CHR of having them?
- Also, CD4 cells/count does not been considered in the Cox model.
- Different studies showed how late-presenters had increased mortality. I believe that the authors should discuss it.

**Authors’ response:**

- The reviewer asked the authors to add data about the cause of death. But, it was already included in the previous manuscript entitled as “Predictors of mortality within one year of ART initiation”.
- As the reviewer stated, both the presence of opportunistic infections at the moment of diagnosis and CD4 cells/count are not presented in the Cox model. This is because of CHR of >0.2 in bi-variable analysis for both variables. As stated in the manuscript, the authors include variables to the Cox model only if the p-value at bi-variable analysis is less than 0.2. That is why these two variables are not included in the Cox model.
- The reviewer asked the authors to discuss how late-presenters had increased mortality. But, it is already discussed in the previous manuscript with a variable name of “pre-ART duration (duration from HIV status confirmation to ART initiation)”.
  - It is also discussed in the document as: *“Similarly, HIV infected patients who started ART six months and above after HIV status confirmation were 2.17 times (AHR=2.17; 95% CI: 1.24, 3.79) at higher risk of death within the first year than patients who started ART early.”*

**Table 1:**

**Reviewer concern**: Please recheck the percentages' approximation. The sum of "age in years", and Educational level" is 100.1% and 99.99% respectively.

**Authors’ response:**

- Thank you for your detail reviewing of this manuscript. The authors revised and corrected it into the correct percentage approximation and the sum became 100% for each variable.

**Reviewer concern**: 67% of people had a baseline CD4 cell count > 200 cells-mL and 41.9 more than 350. It is not clear why 65% of people took cotrimoxazole and 39.47% isoniazid. People who start isoniazid (how long did they take it?) had a positive tuberculin test?

**Authors’ response:**

This is because some patients with confirmed HIV status are hesitated to start ART as early as they know their status. So in order to avoid the occurrence of OIs including TB, CPT was given for HIV patients whatever their CD4 count. Similarly, isoniazid also provided to them after Tb is ruled out.
**Table 2:**

**Reviewer concern**: It is not clear what the authors mean with "Past OI", "Past CPT treatment", "Past INH prophylaxis", and "Past TB treatment history. Do they mean if patients have done the treatments before HIV diagnosis? The authors should include in the table a subtitle to explain all abbreviations used in the table.

**Authors’ response:**

- Past OI = it is to mean presence of opportunistic infection/s after HIV diagnosis and before ART start.
- Past CPT treatment = it is to mean that "does a patient took cotrimoxazole preventive therapy (CPT) after HIV diagnosis and before ART start to prevent the occurrence of OIs"
- Past INH prophylaxis = means that "does a patient took Isoniazid (INH) prophylaxis after HIV diagnosis and before ART start to prevent the occurrence of tuberculosis?" for those who were ruled out from Tuberculosis disease. But, since the study was based on chart review, it was not informative whether TB was ruled out with tuberculin skin test or other tests; rather we simply took the record labeled as “TB status: positive or negative” irrespective of the type of laboratory performed.
- Past TB treatment history = it is to mean that “did a patient took anti tuberculosis treatment before start of ART?”
- In the revised manuscript, a key is included to explain abbreviations in the tables.

**Table 3.**

**Reviewer concern:**

- Please remove "(continued)", or rename the table as table 2.
- About the initial ART regimens, the authors choose as variable "le" and "others". Please correct it.
- Furthermore, I believe that it is interesting knowing what other regimens the patients started. In my opinion, this is important to understand why mortality is higher in "others" group.
- The authors should include in the table a subtitle to explain all abbreviations used in the table.

**Authors’ response**:

- The authors tried to merge the table to one table and removed the term “Continued”.
- In the revised manuscript **1e** is edited as TDF-3TC-EFV and the reason that the authors categorize the initial ART regimens as "le" and "others" is that more than 94% of patients’ records showed that patients had took TDF-3TC-EFV and only 27 patients (5%) took other than TDF-3TC-EFV. Even, this 5% is constituted from the sum of five different combination therapies; namely: AZT + 3TC + EFV, TDF + 3TC + NVP, AZT + 3TC + LPV/r, AZT + 3TC + ATV/r, and TDF + 3TC + ATV/r. When these categories tabulated with the outcome variable, the assumption of chi-square was violated. So, to keep the assumption, the variable was re-categorized by merging these five combinations in to one (i.e. others).
- In the revised manuscript, a key is included to explain abbreviations used in the table and drugs included in “others” category.

**Table 4**

**Reviewer concern:**

- It should be in the results section and not in the discussion
- The authors should include in the table a subtitle to explain all abbreviations used in the table.
- In table 2 the authors divided the educational level into "no education", "primary", "secondary", and college +". In this table, instead, they divided it into "no education", "10 education", and "20 & above". I suggest using the same division.

**Authors’ response**:

- Thank you for the comments; actually it is table 3 not table 4 as I stated it before.
- In the revised manuscript table 3 is sited in the results section, as per the comment.
- Regarding educational level categorization: based on the comment I preferred to use common category in both tables (i.e. "no education", "1^0^ education", and "2^0^ & above") and corrected in the revised manuscript.

**Figure 1**:

**Reviewer concern:** It is not clear what the number between brackets mean. Please comment.

**Authors’ response**:

- The numbers in the brackets in Figure 1 is to show the number of deaths within the specified time interval. For example, number 20 in bracket is to mean that 20 deaths were occurred between time zero and 0.2 years. But, in order to avoid confusion the authors avoid these numbers in brackets at the revised manuscript.

**All figures:**

**Reviewer concern**:

- In the text, the authors wrote about mortality using "months of follow-up," while in the figures, time is expressed as a fraction of years.
- It could create confusion in the readers because "0.6" could be interpreted as "6 months", instead of 7.2 months.
- I suggest modifying the X-axes of the figures
- I suggest performing a Log-rank test to compare two samples' survival distributions in the different KM to see if there is a statistical difference.

**Authors’ response**:

- Thank you for your detailed viewing of the document. Actually, the x-axis of each figure was clearly entitled as “analysis time in year” so that “0.6” can be interpreted as 0.6 year. But to avoid a confusion the x-axis is labeled as “analysis time in month” in the revised manuscript, as per the comment.
- Really, I accept the comment regarding performing a Log-rank test to see if there is a statistical difference between groups. Therefore, log-rank test value is analyzed and incorporated in the revised manuscript for each figure (fig2-5).

***Thank you!!***
