# Supplementary material for: Incidence and predictors of mortality within the first year of antiretroviral therapy initiation at Debre-Markos Referral Hospital, Northwest Ethiopia: A retrospective follow up study
Source: PLoS One. 2021 May 14;16(5):e0251648. doi: 10.1371/journal.pone.0251648 (PMC8121335; doi:10.1371/journal.pone.0251648)
Supplement: Attachment — Submitted filename: point by point response_2.docx [file pone.0251648.s004.docx]

**March 20, 2021**

**PLOS ONE editors**

**Journal of PLOS ONE**

**Dear PLOS ONE editors**

**Subject**: Submission of revised manuscript entitled as ***“Incidence and predictors of mortality within the first year of antiretroviral therapy initiation at Debre-Markos referral hospital, Northwest Ethiopia: A retrospective follow up study*”,** after second revision.

**EMID: 57e8cf222f91fd0f**

Thank you for email dated on Feb 22, 2021, enclosing the reviewer’s comments. We have carefully revised the manuscript and incorporated their comments accordingly. Our responses are given in point-by-point response below.

We hope the newly revised version is suitable for publication and look forward to hearing from you in due courses.

**Sincerely!**

Agazhe Aemro Terefe (Corresponding author)

University of Gondar, College of Medicine and health Sciences, School of Nursing, Department of Medical Nursing.

**Point by point response to reviewers’ comments**

**Part I: Editors’ concerns and response by the authors:**

- Dear editors, the authors would like to thank you for your comment and your concern to this manuscript.
- The authors tried to ensure that the manuscript meets PLOS ONE's style requirements.
- The newly revised manuscript contains:
  - A rebuttal letter that responds to each point raised by the academic editor and reviewer(s).
  - A marked-up copy of the manuscript that highlights changes made to the original version.
  - The new version of the revised manuscript without tracked changes.
- Since it is not applicable, the authors didn’t incorporate the laboratory protocol.

**Part II: Reviewers’ concerns and response by the authors:**

**Reviewer #1:**

Thank you for your in-depth and detailed viewing of the manuscript entitled as “Incidence and predictors of mortality within the first year of antiretroviral therapy initiation at Debre-Markos referral hospital, Northwest Ethiopia: A retrospective follow up study”. Your comments inspires the authors to do more in order to have an interesting paper. We hope this manuscript will be considered until publication.

**Reviewer #2:**

Thank you for your full interest in reading the revised manuscript and for your constructive comments regarding the manuscript. Based on your comments, the authors will tried to fix the remaining issues.

**Reviewer concern:**

- Re-reading the manuscript I found many typos (e.g. Efavirinz).
- Abbreviation should be written entirely in the first appearance in the text, even if used in the abstract.

**Authors’ response:**

- Yes, the Authors accept that there are typing errors in the manuscript and we tried to correct and re-type the error in the newly revised manuscript.
- As per the comment, the authors wrote abbreviations entirely in the first appearance.

**Reviewer concern:**

In the previous revision, I ask the authors to discuss the relationship between late presents and mortality in their cohort. The authors reply, "it is already discussed in the previous manuscript with a variable name of “pre-ART duration (duration from HIV status confirmation to ART initiation)”. However, the duration between HIV confirmation and ART initiation does not concern the "late-presenters status", that it is defined as a CD4 cell count <350cells/mm3, without having opportunistic infections. I suggest the authors read this paper 10.1111/j.1468-1293.2010.00857.x, written by the European Late Presenter Consensus working group.

**Authors’ response**:

- Sorry for the misunderstanding; in the newly revised manuscript, the authors tried to show the relation between late presentation and mortality among HIV-infected patients. Also, it is clearly stated in the introduction part from line 92 to 98 (page 5).

**Reviewer concern:**

In my previous revision, I suggested adding more information about the cause of death in the cohort. The authors replied that “it was already included in the previous manuscript entitled as “Predictors of mortality within one year of ART initiation”.” In my opinion, these results must also be reported in this manuscript because they are crucial for the reader, and he/she does not have to search and read another paper to understand the cause of death.

**Authors’ response:**

- Based on the comment, the authors tried to review different kinds of literatures and identified predictors of early mortality stated by different literatures. The authors incorporated these details in the newly revised manuscript (see line 99 to 105 on page 5).

**Reviewer concern:**

The authors reply satisfactorily about the meaning of past opportunistic infection, past CPT treatment, past INH prophylaxis, past TB treatment history. However, I suggest giving this information not only to me, but also to the reader, adding these explanations in the methods section.

**Authors’ response:**

- Based on the comment, the authors incorporated these definitions to the newly revised manuscript at the method section, particularly at the operational definition (see from line 149 to 156 on page 7-8).

**Reviewer concern:**

The authors reply that the presence of opportunistic infections at the moment of diagnosis and CD4 cells/count are not presented in the Cox model. This is because of CHR of >0.2 in bi-variable analysis for both variables. In my opinion, these results are important and need to be discussed because AIDS-presenters normally have an increased risk of death. Furthermore, I suggest adding all this statistical analysis as supplemental material.

**Authors’ response:**

- Based on the comment, the authors discussed this issue clearly in the newly revised manuscript (see from line 283 to 285 on page 15).
- Furthermore, the authors uploaded all the bi-variable analysis table as supplemental material.

**Reviewer concern:**

The authors wrote that many of the patients had a hesitation on starting the antiretroviral treatments. Could it be due to a lack of communication between the medical staff and the patients? Do you think that longer counseling could reduce this hesitation? Do you normally talk with the patients about U=U (undetectable = untransmittable)? Calabrese et al., in their paper, published in “the lancet HIV, explain why providers should discuss U=U with the patients https://doi.org/10.1016/S2352-3018(19)30030-X. Furthermore, a recent analysis on real-life patients reinforced the validity of this message https://doi.org/10.1097/QAD.0000000000002825. In my opinion, discussing this aspect in the Ethiopian context would increase the value and the originality of the work.

**Authors’ response:**

- Thank you for the link you gave to the authors to read more regarding the issue stated in this part.
- Based on the comment you gave, the authors explained the reason why HIV-infected patients hesitate to start ART as early as possible in Ethiopian context.
- In the revised manuscript, the authors incorporated the implication of counseling and educating patients about U=U (undetectable = un-transmittable). (See from line 59 to 67 on page 3-4).

***Thanks too much!***
